# The Treatment of Disabled Men

**Published:** 1918-11-23

**Authors:** 


### The Treatment of Disabled Men.

It will pay every medical man who has, or is likely to have, disabled men amongst his patients to obtain "Instructions on the Treatment of Disabled Men," recently issued by the Ministry of Pensions. The cost is only twopence, and any bookseller can obtain the pamphlet. The instructions contain a mass of precise information about procedure, fees, special forms of treatment and the ways of getting them, and so on and so forth. It often will happen that a doctor will be asked by a discharged man for information as to where he can obtain a special boot or other surgical appliance, or the doctor may desire to prescribe massage or institutional treatment. If this little pamphlet is at hand much of the information will be immediately available. Under certain conditions special diet can be obtained for a pensioner, or home nursing. It is well to know these things, and to have the reference ready.

